# Multibow: Digital Spectral Barcodes for Cell Tracing

**DOI:** 10.1371/journal.pone.0127822

**Published:** 2015-05-26

**Authors:** Fengzhu Xiong, Nikolaus D. Obholzer, Ramil R. Noche, Sean G. Megason

**Affiliations:** Department of Systems Biology, Harvard Medical School, Boston, 02115, United States of America; NIH/NEI, UNITED STATES

## Abstract

We introduce a multicolor labeling strategy (Multibow) for cell tracing experiments in developmental and regenerative processes. Building on Brainbow-based approaches that produce colors by differential expression levels of different fluorescent proteins, Multibow adds a layer of label diversity by introducing a binary code in which reporters are initially OFF and then probabilistically ON or OFF following Cre recombination. We have developed a library of constructs that contains seven different colors and three different subcellular localizations. Combining constructs from this library in the presence of Cre generates cells labeled with multiple independently expressed colors based on if each construct is ON or OFF following recombination. These labels form a unique "barcode" that allows the tracking of the cell and its clonal progenies in addition to expression level differences of each color. We tested Multibow in zebrafish which validates its design concept and suggests its utility for cell tracing applications in development and regeneration.

## Introduction

Recent developments in genetics and fluorescent protein technology have allowed elegant designs to label and distinguish cells with multiple colors. In Brainbow [1], Cre mediated random recombination on genomic insertions of the Brainbow cassette yields a combinatorial expression profile of different fluorescent proteins (FPs) at different levels. These combinations generate up to ~100 possible visually distinguishable colors for a single cell. This color diversity provides powerful resolution in two main applications: Analyzing detailed organizations of complex structures composed of many cells such as neuronal networks [1–7], and unambiguously identifying cells that share clonal origin [8–11]. The reliance of both purposes on high resolution imaging renders zebrafish an excellent system of choice. For lineage tracing in zebrafish, Brainbow-like approaches serve as an important alternative method to direct imaging based cell tracking which is still challenging for many tissues [12,13]. Successful adaptations of the original Brainbow design in zebrafish have been reported [5,9,11].

The use of multicolor labeling in cell tracing depends on two key properties of the color code generation scheme. First, the diversity of color codes is essential to make an accurate call of clonal vs. non-clonal. The more possible color codes a cell may randomly obtain and the more even the chances of obtaining different color codes, the less likely its non-clonal neighbors will obtain the same color. Second, the stability of identifying color codes is crucial for identifying the same cells and/or clonal progenies over extended periods of time and/or large distances of migration. In the original Brainbow design, color codes depend on different expression levels of a few FPs [9,11]. For example if three FPs are used and five different expression levels can be distinguished then 5^3^ = 125 different "colors" (or barcodes) are possible. This barcode space would be adequate for many applications if all possible codes have the same probability to show up in practice. However, the expression levels of FPs do not normally follow an even distribution, effectively reducing barcode randomness. The original Brainbow system also faces challenges on robustly identifying barcodes. Since incomplete recombination is necessary to maximize barcode diversity, barcode identity can change over time because of continued recombination. Moreover, since the barcode relies on accurately detecting different levels of FP expression, it is sensitive to factors that affect FP signal intensity, such as promoter activity, which often changes as cells adopt different fates [14,15], and imaging conditions such as cell depth which causes signal attenuation or autofluorescence which can cause a false increase. Methods that use multiple independent transgenes or have only one copy of Brainbow transgene are unaffected by intensity level change[16–19]. However, they are very limited in label diversity. These limitations thus restrict the exploitation of the promising potential of Brainbow-based cell tracing.

## Results

### A strategy of combining multiple small ON-OFF switch-like brainbow constructs (Multibow)

**Fig 1:**
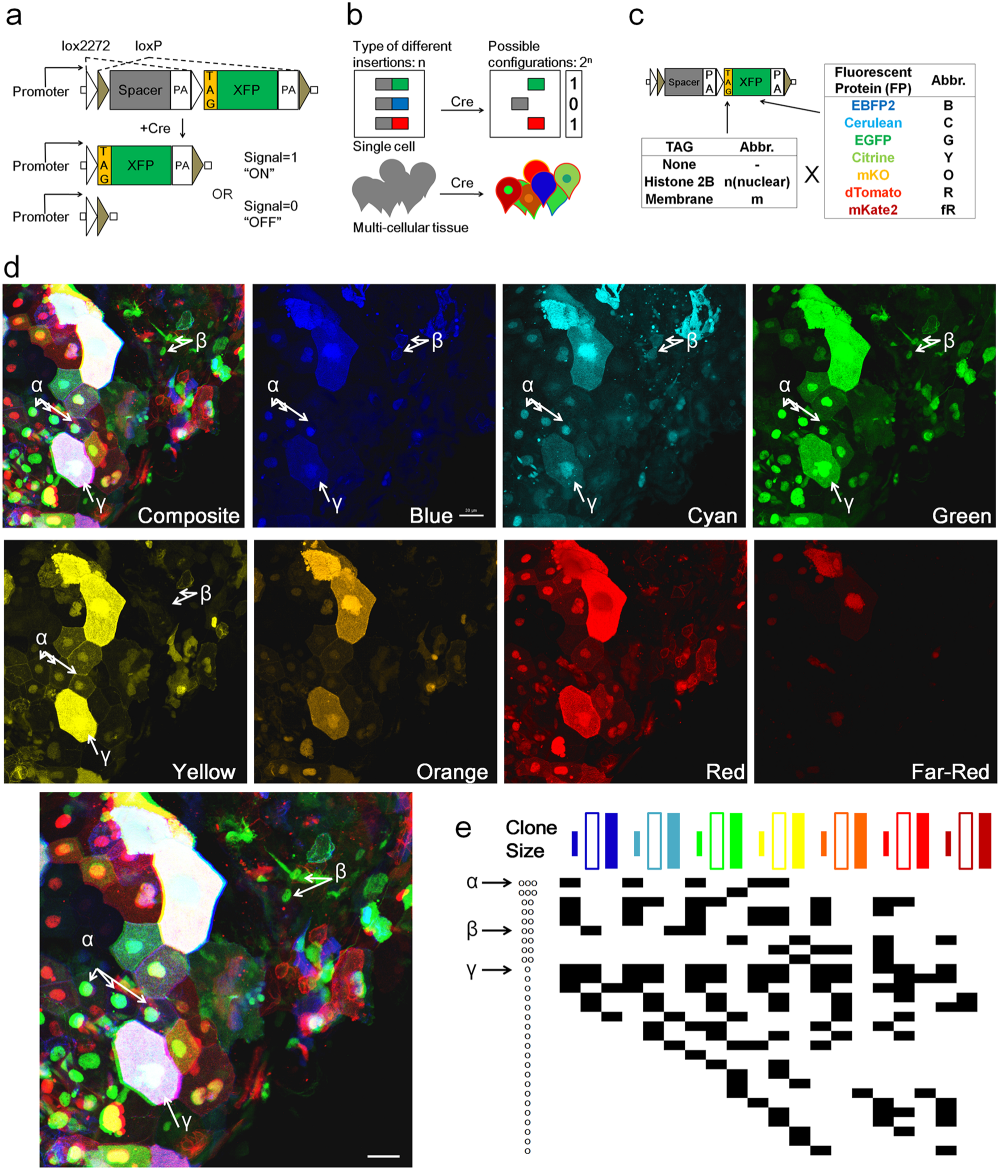
Design and test of Multibow in zebrafish. **a.** Modified “Brainbow [1]” cassette that allows a binary ON/OFF switch. **b.** Multibow Strategy. Each cell harbors multiple different ON/OFF cassettes to generate random color “digital” barcodes upon Cre-mediated recombination. **c.** Table of Multibow Tags and Fluorescent Proteins (FPs). **d.** Diversity of color codes. Image is a densely labeled region along the trunk of a 40hpf *hsp70*:*cerulean-cre* embryo injected with all 21 Multibow constructs and heat-shocked at 10hpf for 1 hour. The color and tag diversity generates barcodes for cell clones that appear random and diverse. Intensity differences further help distinguish cells from neighbors visually. The Composite image is made from the green, yellow (turned to blue) and red panels. 3 different clones are highlighted by α, β, γ and corresponding arrows. Scale bar: 10μm. See also S3 Table. **e.** Partial table of clones of different color codes found in **d.**. The colored square labels of the top row indicate nuclear, membrane and cytoplasmic, respectively. A black square in the table indicates this clone being positive for the corresponding color. Distinct "barcodes" form for different clones. The α, β, γ clones are indicated by arrows. The number of annotated cells labels (~30) represents a large fraction of cells found in the image in **d**, which contains ~50 cells. The fact that most of these cells have a color code distinct from any other cell (except clones that have the same color) show that Multibow label is highly random.

To expand labeling diversity and improve label stability, we modified the original Brainbow design1 to a multiple transgene strategy (Multibow). In Multibow, each FP gene is initially not expressed and then adopts a permanent “ON” or “OFF” status upon Cre-mediated recombination (Fig 1a). Multibow provides theoretically 2^21^ possible “digital” spectral barcodes (Fig 1b) for each single cell by employing 7 FPs of different emission spectra further diversified with 3 different sub-cellular localizations [7,20–22] (21 constructs total, Fig 1c), therefore non-clonally related cells at the time of induction are highly unlikely to arrive at the same color code by chance. To test Multibow in zebrafish, we cloned the transgenes to a *beta-actin2(actb2)* promoter construct carrying Tol2 transposon elements (Fig a in S1 Fig), which cause high frequency genomic insertions of *actb2*:*multibow* upon co-injection of transposase mRNA [23] (Fig b in S1 Fig). We used heat-shock inducible and tissue specific Cre recombinase driver lines (Figs c-d in S1 Fig, data now shown) to activate recombination in injected embryos and take high resolution images of the same live embryos and larvae at different times. Multibow injected embryos show bright, mosaic, multiple colors by 16 hours post heat-shock (Fig e in S1 Fig). Signals of nuclear origin have an oval shape, while the membrane and cytoplasmic signals can be distinguished using confocal slices through the cell (Fig f in S1 Fig).

To assess the extent of color code diversity Multibow can achieve, we examined the emission signal patterns with multiple lasers and filters in embryos injected with all 21 constructs. We found high levels of barcode diversity and randomness (Fig 1d) as predicted by the design. No apparent color code bias or high co-appearance of specific colors were found (Fig 1e) indicating the constructs recombine independently from one another, suggesting high randomness and label diversity. While it is possible to assess the ON/OFF status for all 7 FPs by different lasers, bleed-through signal from other FPs is unavoidable making certain FPs with highly overlapping emission difficult to separate (e.g. OFP and RFP) without careful spectral imaging. In addition, certain FPs (e.g. BFP and CFP) can be masked by autofluorescence in some cell types. In practice, using 4 different color FPs and 4-channel acquisition (e.g. B/G/Y/R, see S3 Table for wavelength ranges), provides simpler and faster imaging set-up and a sufficient barcode diversity (4x3 labels, 2^12^ = ~4k possible barcodes) for most applications. The full set of 7 FPs adds further flexibility to use Multibow with other existing FP markers (e.g. cell specific reporters). In the following sections we use 12 (or less) label injections in the experiments.

### Coverage and stability of Multibow

**Fig 2:**
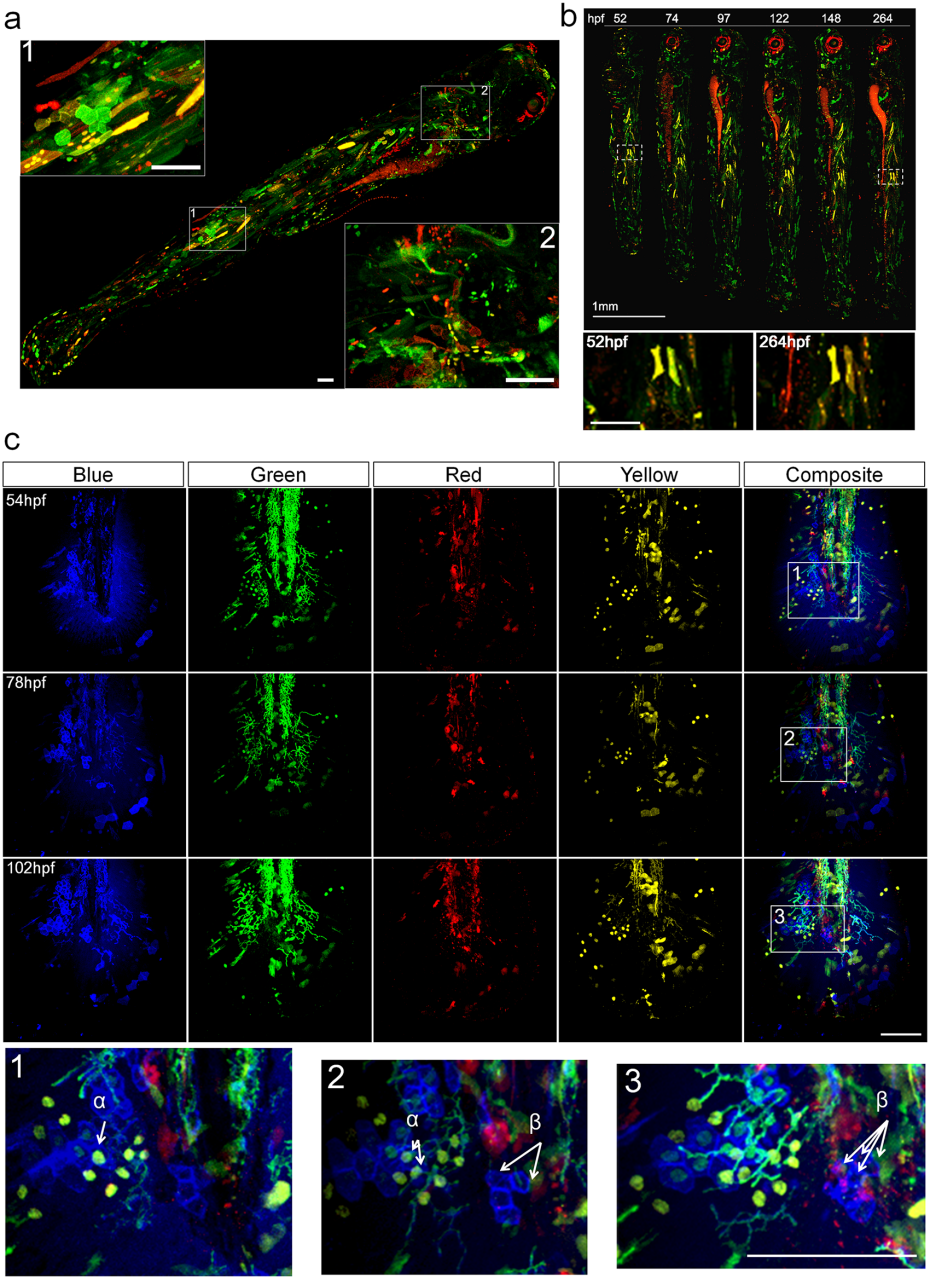
Spatial temporal coverage and stability of Multibow labeling. **a.** Spatial and cell type coverage of Multibow. The embryo was injected with 6 Multibow colors (mR/mG/nR/nG/R/G) at single cell stage and heat-shocked at 1 day-post-fertilization (dpf) for 2 hours. The whole 4dpf larva was imaged in 2 channels (G/R). Positive cells can be seen distributed from head to tail throughout the larva, indicating high spatial coverage. In inserts 1 and 2, distinctly shaped skin, muscle, mesenchymal and neural cells can be observed by cytoplasmic or membrane Multibow labeling. Scale bars: 100μm. **b.** Temporal stability of labeling. The embryo was injected with 6 Multibow colors (mR/mG/nR/nG/R/G) at single cell stage and heat-shocked at 1 day-post-fertilization (dpf) for 2 hours. The same embryo was imaged once per day to 11dpf. The persistence of labeling indicates genomic insertion of Multibow cassettes. Red patches around the eye and along the gut are auto-fluorescence. Enlarged views of white boxed areas show that the area is stably fluorescent. Scale bar in enlarged views: 100μm. **c.** Label stability of color codes over time. The embryo was injected with 12 (B/G/Y/R) Multibow constructs at one cell stage. Heat-shock of this tg(*hsp70*:*cerulean-cre*) individual was at 30hpf (duration: 2 hours). Its developing larval tail fin was imaged every 24 hours starting at 54hpf using four channels (B/G/Y/R). The color codes of the cells remain unchanged despite fluorescent intensity differences at different days, allowing identification of the same cells/clones(e.g., α and β, shown in enlarged regions marked by white boxes). Color codes: α: nG/nY; β: mB. Scale bar: 100μm. See also Fig d in S2 Fig.

Spatially, Multibow cells spread over the whole embryo and show excellent diversity in labeled cell types (Fig 2a). ~25% of embryos show high cell coverage (>15% of cells labeled by Multibow, Figs a-b in S2 Fig). Therefore it is easy to find embryos with dense labeling in tissues of interest from the injected pool of embryos (usually >50 per experiment). Temporally, we found persistence of Multibow expression over 10 days (Fig 2b) indicating stable genomic insertion and a lack of continued recombination of Multibow transgenes, making the strategy feasible to work in older larvae or potentially juvenile and adult animals [23,24].

The design of Multibow suggests that the code should remain the same even if the expression levels of individual FPs change over time or due to imaging conditions such as depth attenuation since the readout for a marker is just ON or OFF rather than quantitative levels. This temporal stability of labeling is crucial to the accuracy of multicolor lineage tracing [11]. We tested this by following Multibow cells closely over the course of 3–5 days (Fig 2c, Figs c-d in S2 Fig). The labeled cells indeed show intensity changes of expression between days but the color “codes” determined by quantizing the presence or absence of individual tagged FPs are unchanged allowing the same cells to be identified. In clones, daughter cells inherit the color codes of mother cells so that their lineage relationship can be identified (Fig 2c). In addition, the color codes stay unchanged upon a second heat-shock induction of Cre (Fig e in S2 Fig). These results show that the design of Multibow can provide stable and discrete color codes for identifying the same cells/clones over time. It is important to note that correctly judging the presence vs. absence of the color is necessary for reliable code identification. In situations where the expression level or a particular Multibow transgene is too low a color coding error might occur.

To evaluate the potential of using Multibow in stable transgenic lines, which would have a higher percentage of labeled cells, we identified founders harboring single or multiple Multibow transgenes. The labeling after recombination was consistent with the design (Fig a in S3 Fig, data not shown), indicating Multibow transgenes can be used in stable transgenic lines. However, we found while some transgenic lines exhibit random color codes (Fig b in S3 Fig), other lines generate unbalanced and/or reduced diversity in color codes (Figs c-d in S3 Fig). These results suggest that Multibow transgenic lines have higher cell coverage and may be useful if many individual Multibow transgenes recombine randomly. The injection strategy, on the other hand, is advantageous given its ease, flexibility, and efficiency despite lower coverage.

### Examples of Multibow applications

**Fig 3:**
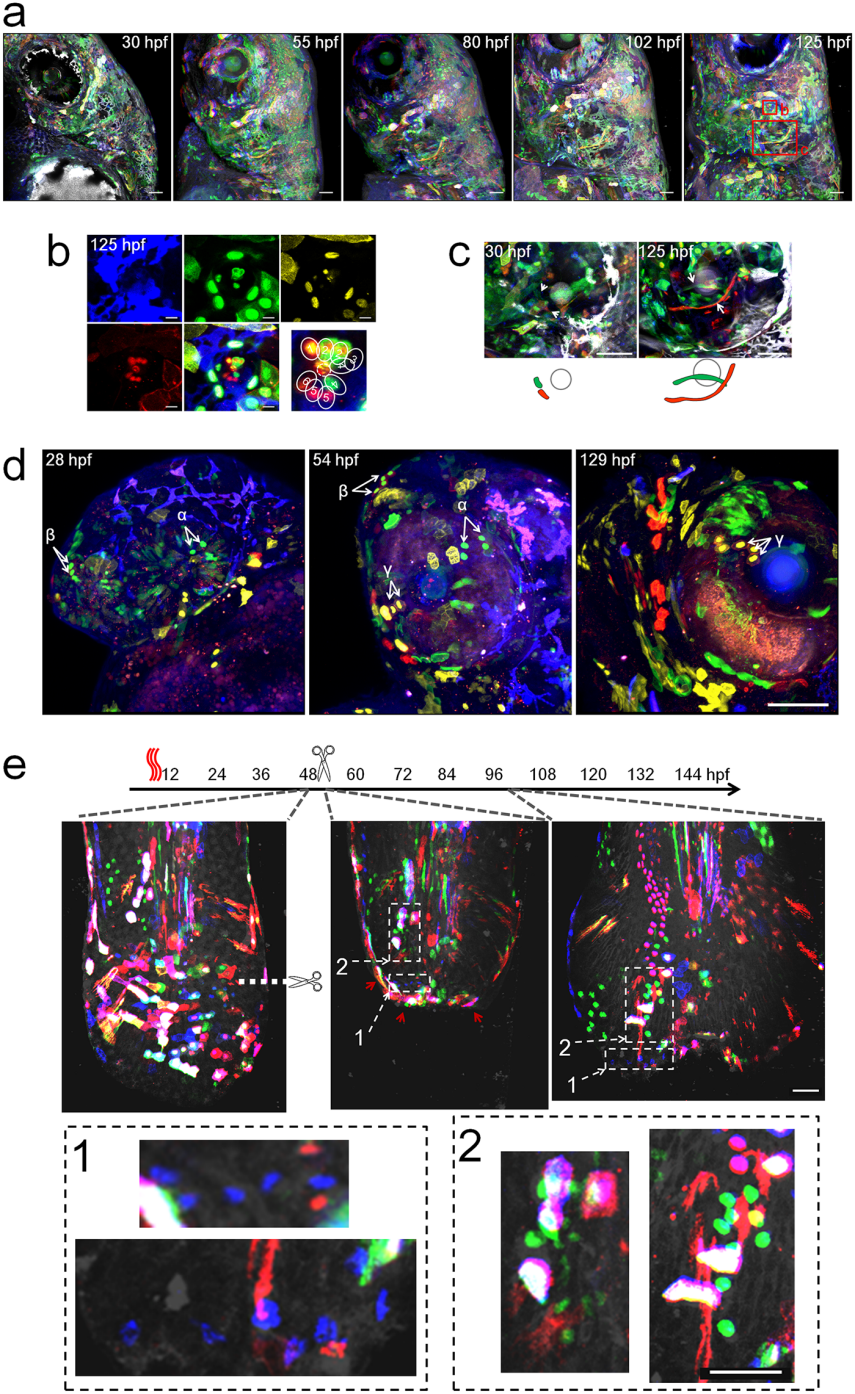
Examples of Multibow Cell Tracing in Development and Regeneration. **a.** Cranial facial development mapped by Multibow. The embryo was heat-shocked at 6hpf. 4 channels (B/G/Y/R) were used. The left face of the larva was imaged. Red boxes: regions highlighted in **b.** and **c.**. Scale bars: 50μm. **b.** Lineage relationship between neuromast hair cells. Dashed line circle indicates the hair bundle. Multibow labeled hair cell color codes: 1(mB/nY/R), 2(mB/mG/nR), 3(mB), 4(nG), 5(mB/nR), 6(R). The same pattern was already observed at 30hpf. Scale bars: 10μm. **c.** Identification of cells that undergo remarkable morphological changes during semicircular canal formation. Arrows: initial locations of the two mesenchymal cells that span the projection later. Grey circle: posterior otolith. Scale bars: 50μm. **d.** Clonal expansion near the eye over long time periods. The embryo was injected with 12 constructs (B/G/Y/R) and heat-shocked at 10hpf. Arrows indicate locations of identified clones α (nG), β (nG/R), γ (nY/mR). These clones can be seen amplified in number at 54hpf or 129hpf (α: 2 to 4; β: 2 to 4; γ: 2 to 3). Scale bar: 100μm. **e.** Multibow analysis of regeneration in the larval tail. Heat-shock labeling (1 hour), amputation and imaging were performed as labeled in the timeline. Immediately after amputation, the tissue shrank and cells near the wound converged (the images overlay may appear to be slightly out of register due to the changes of the live tissue during the acquisition of different channels, cell identification is not affected as these changes are small and predictable). By 2 days after amputation, most cells that had converged at the frontier of the wound were gone (their unique color codes disappeared, red arrowheads). The regenerated tissue came from clonal expansion of cells away from the frontier (highlighted examples in enlarged view from the white boxes 1 and 2). These clones show lineage restriction to the original cell type (the morphology of cells in the same clone remains similar, e.g., the blue cells in box 1 increased in number while size and shape do not have major changes.). Scale bars: 50μm.

We used Multibow to map craniofacial development of a single larva through time by taking one image stack per day (Fig 3a). Many cells and clones are traceable over a 5-day period, an interval that is not currently feasible for performing direct timelapse imaging. The images reveal details of the cell influx that populates the facial structures. We highlight two regions of the “facemap” as examples. In one neuromast (Fig 3b), we find that some hair cells share the exact same color code (e.g., the two cells labeled by "2" share the color code mB/mG/Y/nR), suggesting that they share a lineage, i.e. progenitors undergo symmetric divisions to give rise to two hair cells. Other hair cells are uniquely coded. These results are consistent with previous work using complex live imaging systems [25]. In the inner ear (Fig 3c), where extensive morphogenesis movements build the semi-circular canals, we identified initial locations of cells that underwent drastic elongation (>100μm) across the length of the projections (arrowheads). With Multibow we could exclude other cells in the neighborhood. The complex morphological changes of these cells make it challenging to discover them with ubiquitous markers or to trace them with timelapse imaging. In a developing eye (Fig 3d), we are able to identify and follow clones over extended periods of time using a minimal number of acquisitions, even though the tissue has grown considerably and cell locations have changed significantly. For example, the cells labeled as clone "γ" have a code of nY/mR and are the only cells in the tissue area to have this code at 54hpf. By 129hpf, a group of cells with the same code is found and can confidently be identified as the same cells. We also used Multibow to analyze the cellular origins of regenerated tissue, using the larval tail as an example (Fig 3e). Larvae with a suitable number of bar-coded cells in the tail tip were selected and subjected to imaging before, immediately after and 2 days after amputation. The regenerated tissue contains amplified clones of cells of restricted lineages as identified by the same color codes and morphologies, consistent with experiments that use single color mosaic labeling in a large number of animals [24].

## Discussion

Our tests of Multibow in zebrafish suggest the potential wide application of this design in questions involving cell tracing. The concept of Multibow brings two improvements to the Brainbow-based multicolor labeling strategy. First, the use of a wider range of FPs and different sub-cellular localization tags allows for the creation of a much larger barcode space, which importantly should all be accessible with similar probabilities by Cre recombination since the constructs are all similar. Second, the use of an ON/OFF indicator makes the color codes more robust to changes in hue, saturation and intensity, which have been the basis for color-codes in previous Brainbow approaches but can be confounded by differential expression, maturation, bleaching, or depth-attenuation of fluorescent labels.

The full potential of Multibow is yet to be realized. First, imaging acquisition and analysis using the full set of 21 constructs are non-trivial given the overlapping emission spectra of the FPs. Second, in the injection of Multibow in zebrafish, the cell divisions that follow cause uneven inheritance of the injected pool, which makes the labeling mosaic. While mosacisim adds an advantage for lineage tracing as more separated clones are easier to identify and follow, it reduces the total fraction of labeled cells. Less information can be recovered than in densely labeled embryos so more embryos are needed obtain statistical lineage information. Third, a delay of labeling onset exists between the induction of Cre activity and detectable fluorescence, during which the cell may divide, so it is not practical to be exact on when the cell/clone obtains its barcode. This delay could be potentially shortened by using drug inducible Cre but expression and maturation of the FP will still take some time. Fourth, in certain cell types or situations the sub-cellular localization tags may become difficult to separate. For example, in cells with a bright cytoplasmic signal and weak membrane signal of the same FP, the membrane signal may not be distinguished during analysis. If this relative brightness changes over time, a barcoding error may appear. These specific cases should be considered in Multibow imaging analysis to ensure validity of cell/clone identification.

Despite the discussed limitations, Multibow has a clear potential to scale up for high-throughput mapping of development and regeneration by using automated image analysis approaches [26], and is also adaptive for use with different promoters, Cre drivers and transgenic markers that are available to study specific problems. Multibow is also potentially useful in other model organisms where high genomic insertion rate is technically achievable [27,28]. Genomic editing strategies will likely help generating Multibow transgenic organisms with controlled precision. We believe the power and applicability of Multibow are likely to quickly increase in the near future following the continued rapid advances in genome editing, fluorescent proteins, and imaging technologies.

## Materials and Methods

### Multibow cloning and preparation

To clone Multibow constructs, the original pCMV-Brainbow-1.1 [1] construct was digested with NheI/XmnI and the 3.4kb fragment was cloned into a pMTB vector containing the Tol2 elements and the beta-actin2 (*actb2*) mini gene driver [13] (Fig a in S1 Fig) at the SpeI/ZraI sites. The resulting construct was digested with BstBI/BbsI and re-ligated to truncate both the original mem-cherry and Kusabira orange sequences to generate the non-fluorescent spacer sequence flanked by loxP and lox2272 sites. The resulting construct served as backbone for cloning of different FP sequences at the original mem-EYFP site. To facilitate modularity and efficiency of cloning, small linker sequences were used to connect the backbone to different tags/FPs and between tags and FPs. Primers joining linker sequences and FP/backbone sequences (See S1 Table for the list of FPs used and other sequences) were used to amplify the vector, FPs and tag fragments using a Fusion High-Fidelity PCR Mix (NEB). Fragments were combined in an isothermal assembly reaction [29] (S2 Table) to generate Multibow constructs. Reaction mixes were transformed with 5-alpha F'Iq cells (NEB) and screened by colony PCR. Multibow constructs displayed instability in *E*.*coli* cultures and had low yield in Maxi/Midi-preps. Mini-prep was used to harvest Multibow constructs (Resulting yield averaged at 1.5–2μg per Multibow construct and was sufficient for many experiments (See below for the injection protocol)). A further purification step using the MinElute PCR purification kit (Qiagen) was applied to clean and concentrate Multibow constructs. Using isothermal assembly, the Multibow cassettes can be efficiently cloned into other vector backbones. To generate the *elavl3*:*cerulean-cre* construct for transgenic lines, the elavl3(HuC) promoter [30] was cloned to replace the actb2 promoter in an *actb2*:*cerulean-cre* construct. To generate the *hsp70*:*cerulean-cre* construct for transgenic lines, the *hsp70* promoter was cloned to replace the actb2 promoter in a *actb2*:*cerulean-cre* construct. The Cerulean-Cre fusion protein allows real time monitoring of presence of Cre in live embryos.

### Zebrafish maintenance, injection and transgenic lines

All fish are housed in fully equipped and regularly maintained and inspected aquarium facilities. Zebrafish work was approved by the Harvard Medical Area Standing Committee on Animals under protocol number 04487. AB wild-type and RNF pigmentation mutant strains were used. The transgenic strains (tg(*elavl3*:*cerulean-cre*), tg(*hsp70*:*cerulean-cre*) and tg(*actb2*:*multibow*) lines) were generated by standard injection/screening methods. Natural spawning was used to obtain embryos and time of fertilization was recorded according to the single cell stage of each clutch. Embryos were incubated in 28°C during imaging and all other times except room temperature during injections and dechorionating. To make the transposase mRNA, a pCS-TP [19] construct was linearized with NotI and purified with the MinElute PCR purification kit (Qiagen). The construct was then used to synthesize transposase mRNA with an in vitro transcription system (mMESSAGE mMACHINE, Ambion). The injection solution contained 30ng/μl transposase mRNA and 20ng/μl mixture of Multibow constructs (equal quantity of each single construct). The combinations of Multibow constructs were chosen depending on experimental needs. 1-cell stage (1-cell stage is important for good labeling coverage) embryos were injected with a micro-injection apparatus (Nanoject) with 2.3nl injection solution and were screened (MVX fluorescent macro scope, Olympus) for health before applying heat shock (37°C air incubator, timing and duration depend on experimental needs). The injected embryos show slight delay of development as expected from injection experiments. No significant health difference was observed between Multibow injected embryos and control embryos. Amputation of the tail was performed using a clean razor blade on anesthetized (0.2% Tricaine) embryo/larva on agarose mounts.

### Imaging and image analysis

Embryos showing Multibow expression were screened and selected with MVX fluorescent macro scopes (Olympus) and then mounted to imaging molds as described [12,31]. Z-stacks were taken using a Zeiss 710 confocal microscope and Zen software with a home-made heating chamber maintaining 28°C. Different laser and filter settings were used for different FPs (S3 Table). Bleed through signal is common with FPs that have overlapping emission spectra, but can be distinguished based on signal pattern in adjacent channels using a different excitation laser and/or filter. Barcodes were assigned based on the presence/absence of signal in individual channels. Single channel original images were analyzed in Zen (Zeiss) through Z-slices and rendered into 3D composite views with FluoRender 2.10 (http://www.sci.utah.edu/software/127-fluorender.html). The 3D images were further overlaid in Photoshop to make composite multicolor figures.

## Supporting Information

S1 Fig Design and Validation of Multibow. **a.** Multibow construct map for use in the Zebrafish system. Example construct is pMTB-Multibow-fR(mKate2). Red box: variable region where membrane and nuclear tagged versions are different from the map. See sequence files for details. See also S1 Table. **b.** Mosaic and uneven distribution of injected DNA. Embryos were injected at 1-cell stage with 20ng/μl pMTB-citrine DNA construct. Yolk is bright with autofluorescence. **c.** Analysis of Cre level dynamics in tg(*hsp70*:*cerulean-cre*). SC, spinal cord. M, muscle. 0.5h heat-shock does not induce significant Cerulean-Cre expression. Muscle cell expression is more sensitive to heat-shock. Low level expression is also present in some muscle cells without heat-shock. 1.0h heat-shock provides an optimal pulse. Scale Bar: 10μm. **d.** Average fluorescent intensities (+/- s.d.) measured in 10 nuclei from the images in **c. e.** Onset of Multibow after heat-shock induced Cre expression. The whole pool of 21 constructs were injected. The time required for Multibow to become detectable after Cre induction limits its application for lineage tracing in early stage zebrafish embryos before 20 hours post fertilization (hpf). However, the early stages are often more feasible for direct live imaging based lineage analysis [12,13,31]. **f.** Distinction of membrane and cytoplasmic signals. In 3D projection images (most of the figures) it may be difficult to distinguish signals of membrane and cytoplasmic origin, as bright membrane signal is often detectable in cytoplasm and in 3D overlay the signals overlap. Looking through original confocal slices removes most of the difficulties. The top images show a comparison of membrane signal in 3D rendering and slice view, and a slice view of a cytoplasmic signal. The main distinction is a bright edge and fuzzy cytoplasmic signal for membrane FPs and a homogenous signal throughout for untagged FPs. Bottom images show the contrast of neighboring cells with membrane labeled vs. untagged FPs of the same color. Difficulties may rise when trying to distinguish the presence of both membrane and cytoplasmic signals of the same FP when the membrane signal is much brighter (See Discussion).
(TIF)

S2 Fig Coverage and Stability of color codes. **a.** Cell fraction coverage of Multibow analyzed by co-injecting the complete Multibow cocktail with mem-cherry mRNA. The presence of a strong Red ubiquitous marker (mem-cherry) excludes labeled cells by mR/R to be identified. The actual fraction using all Multibow channels may be higher. The coverage is categorized as "high", "medium" and "low" depending on the fraction of cells labeled. Scale Bar: 10μm. **b.** Summary of injected embryos by fraction of Multibow labeled cells as categorized in panel a. 1-Cell stage injection is critical for higher fraction of labeled cells. **c.** (i) Example cells in which background intensity in Blue channel has dropped while nG intensity increased. (ii) Example cell in which both total and relative intensity of FPs have changed. These cells appear to have changed “color” but can be identified based on the unchanged ON/OFF status of individual tagged FPs. Pixel channel intensity distribution of cells were further analyzed in down-sampled images of 1/8 original pixel number (down-sampling was used to reduce the number of pixels to analyze). The RGB intensity values of pixels belonging to the cells were plotted for both time points. The colors of the same cell(s) may change as a result of signal changes between different acquisition times, as reflected by a shift of pixel distribution in the RGB color space. The cells were from experiments in panel e. **d.** Label stability of color codes to intensity changes. A lateral region of a group of labeled muscle cells were followed for 5 days after injection of 12 Multibow constructs (R/G/Y/B) and heat-shock for 1 hour at 22hpf. The expression profiles of the same cells are invariant during this time despite intensity fluctuations. Four channels were used. Note the 24hpf blue image shows ubiquitous labeling of nuclei by the heat-shock induced Cre-Cerulean expression in this tg(*hsp70*:*cerulean-cre*) individual, which fades away afterwards (See also Fig c in S1 Fig). The enlarged merged views show an example cell that apparently changed color due to intensity fluctuations but kept the same Multibow barcode. Scale bars: 10μm. **e.** Intensity fluctuation tests. The embryos were injected with 6 constructs (B/G for the upper panel and G/R for the lower panel, respectively). Heat-shock labeling and imaging were performed as labeled in the timeline. The tissue undergoes growth, and clonal expansion of labeled cells is evident (boxes in the upper panel). The color codes remain unchanged (e.g. arrowheads in the lower panel) even though the intensities of expression may change (boxes in the upper panel). No new color codes appear after the second heat-shock, indicating completion of recombination by the first heat-shock. Scale Bar: 100μm.
(TIF)

S3 Fig Analysis of Multibow transgenic lines. **a.** Validation of a single color Multibow line (*tg(actb2*:*multibow(nO)/+)*). Multibow transgenic lines were crossed with a Cre driver line as indicated on the top (Same for following panels). Multibow cells were scored to determine the onset of Multibow labeling after Cre addition and distribution of color codes. HS, heat-shock at indicated times (duration: 3 hours). **b.** A double color Multibow line (*tg(actb2*:*multibow(mYnO)* ^*1*^ *)*) showing ~60% cell coverage and bias towards more nO+ cells. Superscript indicates line number from independent founders. Arrows, example of each of the 4 color codes. Image is a lateral confocal slice of the hindbrain, 2dpf. All scale bars: 10μm. **c.** Another double color Multibow line (*tg(actb2*:*multibow(mYnO)* ^*2*^ *)*) showing mY+ cells cover all nO+ cells, the code of mY/nO+ is lost in this line. Arrows: mY+/nO- cells. Image is a lateral confocal slice of the trunk, 2dpf, heat-shocked at 12hpf. **d.** This line (same as in panel c.) shows very strong coverage of mY+ cells (few mY- cells when Cre expression is constitutive), reducing diversity of color codes. Image is a lateral optical slice of the brain, 2dpf.
(TIF)

S1 Table Sequences Table. Includes primer sets, tag sequences and FP sequences used to assemble Multibow constructs. See also sequence files, S1a Fig Notes on FP sequences: Cerulean, citrine and mKO were codon modified sequences that contained silent mutations to decrease homology among FP variants.
(XLSX)

S2 Table Modified Isothermal Assembly Ingredients Table. Mix fragments at equal molecular ratio in 5μl and mix with 1 Isothermal Assembly reaction aliquot (15 μl), then incubate at 50°C for 15–30 minutes. Use 1μl reaction mix to transform competent cells.
(XLSX)

S3 Table Confocal Imaging Settings Table. Applying additional laser lines at different wavelengths will help distinguish the presence of different FP genes. See also Figs 1d–1e.
(XLSX)
