# Supplementary figures and images for: Multibow: Digital Spectral Barcodes for Cell Tracing
Source: PLoS One. 2015 May 26;10(5):e0127822. doi: 10.1371/journal.pone.0127822 (PMC4444339; doi:10.1371/journal.pone.0127822)

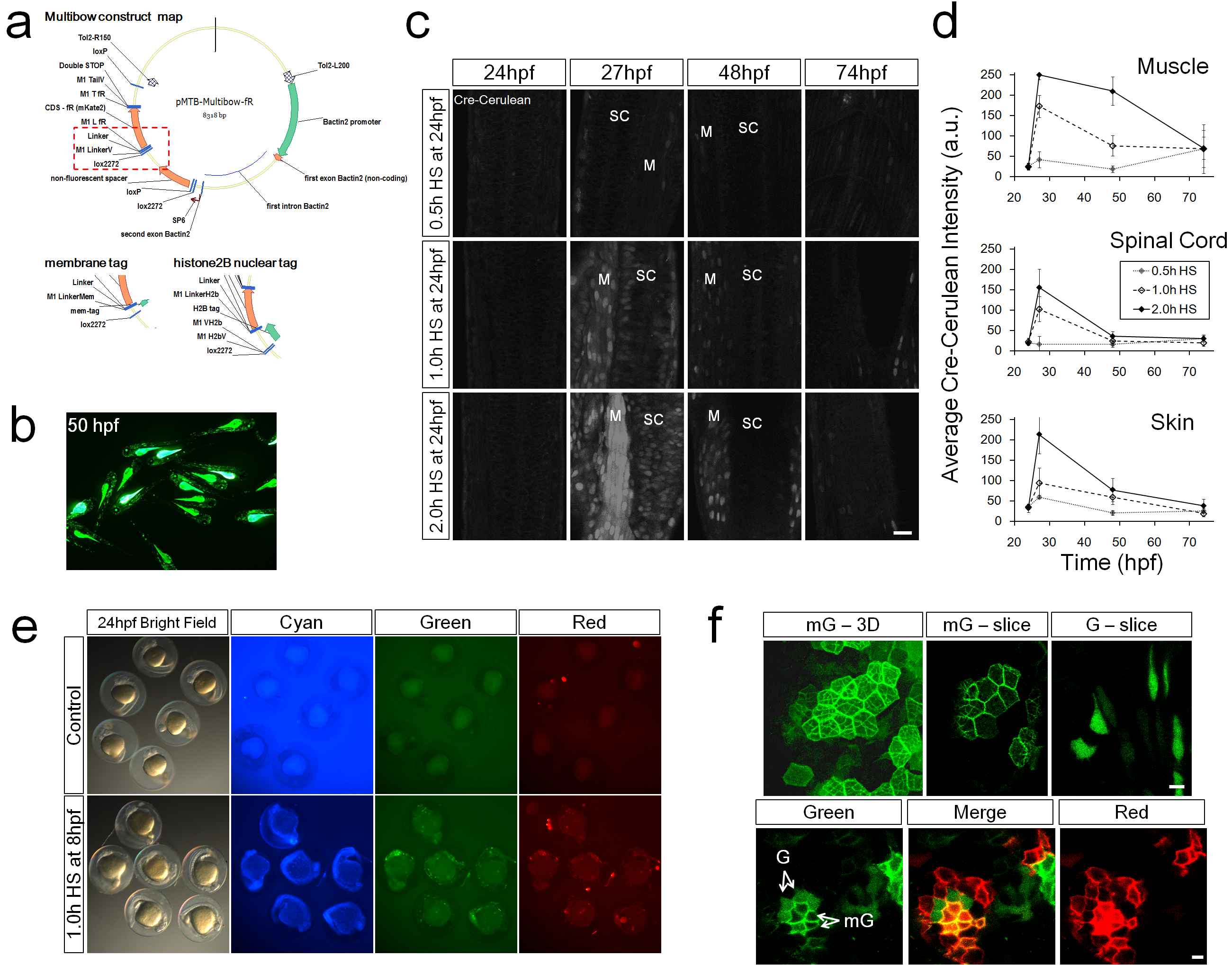

Supplement: S1 Fig — a. Multibow construct map for use in the Zebrafish system. Example construct is pMTB-Multibow-fR(mKate2). Red box: variable region where membrane and nuclear tagged versions are different from the map. See sequence files for details. See also S1 Table. b. Mosaic and uneven distribution of injected DNA. Embryos were injected at 1-cell stage with 20ng/μl pMTB-citrine DNA construct. Yolk is bright with autofluorescence. c. Analysis of Cre level dynamics in tg(hsp70:cerulean-cre). SC, spinal cord. M, muscle. 0.5h heat-shock does not induce significant Cerulean-Cre expression. Muscle cell expression is more sensitive to heat-shock. Low level expression is also present in some muscle cells without heat-shock. 1.0h heat-shock provides an optimal pulse. Scale Bar: 10μm. d. Average fluorescent intensities (+/- s.d.) measured in 10 nuclei from the images in c. e. Onset of Multibow after heat-shock induced Cre expression. The whole pool of 21 constructs were injected. The time required for Multibow to become detectable after Cre induction limits its application for lineage tracing in early stage zebrafish embryos before 20 hours post fertilization (hpf). However, the early stages are often more feasible for direct live imaging based lineage analysis [12,13,31]. f. Distinction of membrane and cytoplasmic signals. In 3D projection images (most of the figures) it may be difficult to distinguish signals of membrane and cytoplasmic origin, as bright membrane signal is often detectable in cytoplasm and in 3D overlay the signals overlap. Looking through original confocal slices removes most of the difficulties. The top images show a comparison of membrane signal in 3D rendering and slice view, and a slice view of a cytoplasmic signal. The main distinction is a bright edge and fuzzy cytoplasmic signal for membrane FPs and a homogenous signal throughout for untagged FPs. Bottom images show the contrast of neighboring cells with membrane labeled vs. untagged FPs of the same color. Diffi [file pone.0127822.s001.tif]

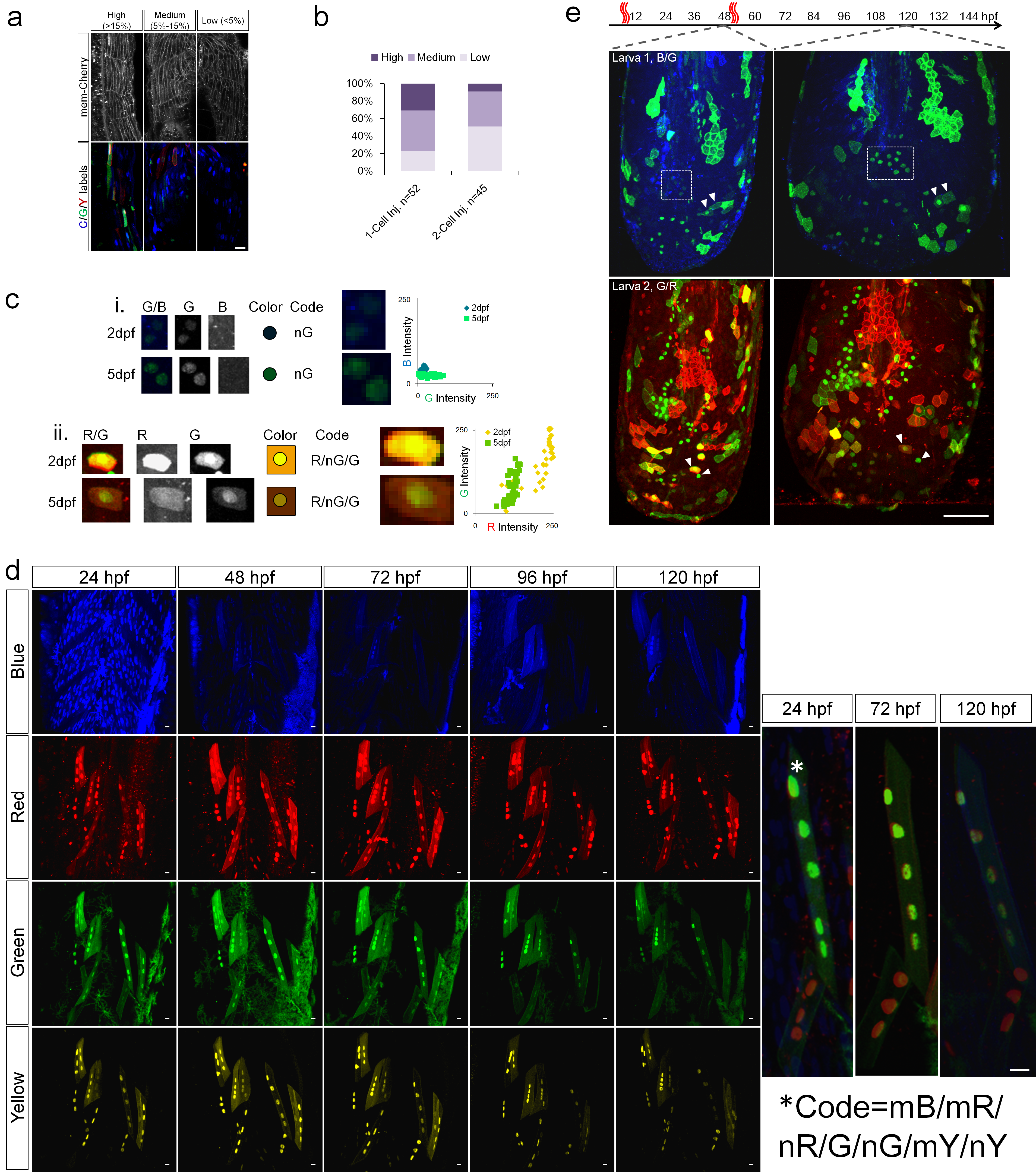

Supplement: S2 Fig — a. Cell fraction coverage of Multibow analyzed by co-injecting the complete Multibow cocktail with mem-cherry mRNA. The presence of a strong Red ubiquitous marker (mem-cherry) excludes labeled cells by mR/R to be identified. The actual fraction using all Multibow channels may be higher. The coverage is categorized as "high", "medium" and "low" depending on the fraction of cells labeled. Scale Bar: 10μm. b. Summary of injected embryos by fraction of Multibow labeled cells as categorized in panel a. 1-Cell stage injection is critical for higher fraction of labeled cells. c. (i) Example cells in which background intensity in Blue channel has dropped while nG intensity increased. (ii) Example cell in which both total and relative intensity of FPs have changed. These cells appear to have changed “color” but can be identified based on the unchanged ON/OFF status of individual tagged FPs. Pixel channel intensity distribution of cells were further analyzed in down-sampled images of 1/8 original pixel number (down-sampling was used to reduce the number of pixels to analyze). The RGB intensity values of pixels belonging to the cells were plotted for both time points. The colors of the same cell(s) may change as a result of signal changes between different acquisition times, as reflected by a shift of pixel distribution in the RGB color space. The cells were from experiments in panel e. d. Label stability of color codes to intensity changes. A lateral region of a group of labeled muscle cells were followed for 5 days after injection of 12 Multibow constructs (R/G/Y/B) and heat-shock for 1 hour at 22hpf. The expression profiles of the same cells are invariant during this time despite intensity fluctuations. Four channels were used. Note the 24hpf blue image shows ubiquitous labeling of nuclei by the heat-shock induced Cre-Cerulean expression in this tg(hsp70:cerulean-cre) individual, which fades away afterwards (See also Fig c in S1 Fig). The enlarged merged views show an examp [file pone.0127822.s002.tif]

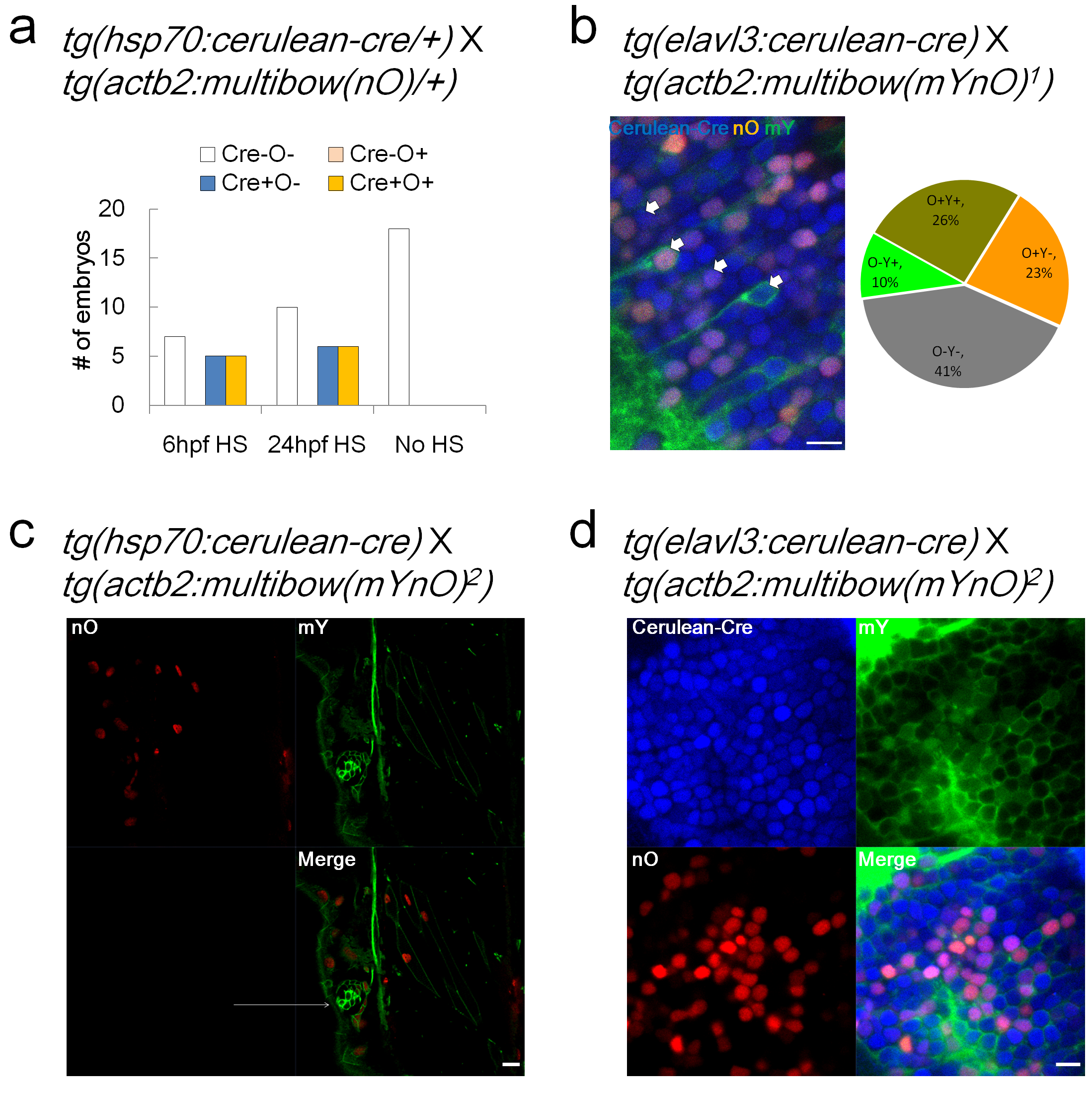

Supplement: S3 Fig — a. Validation of a single color Multibow line (tg(actb2:multibow(nO)/+)). Multibow transgenic lines were crossed with a Cre driver line as indicated on the top (Same for following panels). Multibow cells were scored to determine the onset of Multibow labeling after Cre addition and distribution of color codes. HS, heat-shock at indicated times (duration: 3 hours). b. A double color Multibow line (tg(actb2:multibow(mYnO) 1 )) showing ~60% cell coverage and bias towards more nO+ cells. Superscript indicates line number from independent founders. Arrows, example of each of the 4 color codes. Image is a lateral confocal slice of the hindbrain, 2dpf. All scale bars: 10μm. c. Another double color Multibow line (tg(actb2:multibow(mYnO) 2 )) showing mY+ cells cover all nO+ cells, the code of mY/nO+ is lost in this line. Arrows: mY+/nO- cells. Image is a lateral confocal slice of the trunk, 2dpf, heat-shocked at 12hpf. d. This line (same as in panel c.) shows very strong coverage of mY+ cells (few mY- cells when Cre expression is constitutive), reducing diversity of color codes. Image is a lateral optical slice of the brain, 2dpf. (TIF) [file pone.0127822.s003.tif]
